# AI‐assisted software development for brachytherapy seed inventory management

**DOI:** 10.1002/acm2.70795

**Published:** 2026-09-09

**Authors:** Tonghe Wang, Joel Beaudry, Antonio L Damato, Marisa Kollmeier, David Aramburu Núñez

**Affiliations:** ^1^ Department of Medical Physics Memorial Sloan Kettering Cancer Center New York New York USA; ^2^ Department of Radiation Oncology Memorial Sloan Kettering Cancer Center New York New York USA

**Keywords:** AI assisted, brachytherapy, in‐house software, vibe coding

## Abstract

**Purpose:**

Medical physics departments frequently rely on in‐house software to address niche clinical needs, yet these tools are often built by physicists without formal software engineering training, resulting in legacy applications that are difficult to maintain, poorly documented, and vulnerable to obsolescence from operating system upgrades. This study investigates the use of AI‐driven “vibe coding”, i.e. software development through natural language prompting, to enable a clinical physicist with no web development experience to replace an obsolete MATLAB‐based radioactive seed inventory system with a modern desktop application.

**Materials and methods:**

Development was performed using Claude Code (Anthropic) powered by the Claude Sonnet 4.6 large language model. A structured “Plan‐Review‐Execute‐Verify‐Feedback” (PREVF) methodology governed all development, with the physicist providing natural language prompts and the AI generating all source code. The resulting application manages five seed models across four clinical applications, with features including seed batch lifecycle tracking, calibration record management, audit logging, waste deposit tracking, etc. Evaluation metrics included development time and prompt volume, feature completeness against eight pre‐defined requirements, System Usability Scale (SUS) scores comparing the legacy and new systems, and an independent maintainability test in which team members with minimal coding experience attempted modifications without developer assistance.

**Results:**

The application was developed in approximately 13 hours, using approximately 115 natural language prompts. All eight functional requirements were fully implemented. The new application received a median SUS score of 95 compared to 32.5 for the legacy system. In the maintainability evaluation, three independent testers completed bug fixes and feature modifications in one to two prompts each, all in under 10 min, without guidance from the original developer.

**Conclusion:**

AI‐driven vibe coding enables clinical medical physicists to rapidly develop and maintain operational software, resolving the maintenance bottleneck characteristic of legacy in‐house tools. The PREVF methodology provides a structured framework for physicist oversight of AI‐generated code. This approach has potential applicability to provide clinical support tools by medical physics departments.

## 1 INTRODUCTION

Clinical medical physics requires a wide range of operational and quality assurance tasks that require specialized software support. While commercial systems address core functions such as treatment planning and record‐and‐verify, many ancillary tasks including machine QA analysis, independent dose calculation, plan checking and inventory management remain without adequate commercial solutions, particularly for smaller or more specialized clinical programs. As a result, medical physics departments have historically relied on in‐house developed software to fill these gaps. This challenge is especially acute in brachytherapy, a highly specialized modality that serves a relatively small patient population compared to external beam radiotherapy. The limited clinical volume and highly institution‐specific workflows of brachytherapy programs provide little financial incentive for commercial vendors to develop dedicated ancillary tools, leaving tasks such as radioactive seed inventory management, source tracking, and disposal documentation almost entirely dependent on locally built solutions.^1^, ^2^, ^3^, ^4^, ^5^, ^6^

The development of such in‐house tools has traditionally fallen to clinical physicists themselves, who possess deep domain knowledge of the clinical problems but often lack formal training in modern software engineering practices and user interface/user experience (UI/UX) design.^7^ Constrained by heavy clinical workloads that leave limited time for software development, physicists have commonly built tools using platforms with low barriers to entry, such as MATLAB (MathWorks, Natick, MA) and Microsoft Excel. While functional at the time of their creation, these tools frequently accumulate technical debt over years of use: they become difficult to maintain as the original developer moves on, challenging to extend as clinical needs evolve, and frustrating for end users due to outdated interfaces and poor usability. Compounding the problem, mandatory operating system upgrades driven by institutional IT security policies can abruptly render legacy software non‐functional when newer versions of Windows deprecate runtime environments, libraries, or APIs on which these tools depend. This “maintenance bottleneck” where only the original author can effectively modify the software represents a systemic vulnerability in clinical physics departments.

Recent years have seen growing recognition of the need for dedicated software engineering support within medical physics teams. A recent study demonstrated that integrating even a part‐time software engineer into a medical physics department significantly improved workflow efficiency, research capacity, and system integration.^7^ However, hiring dedicated software engineers remains impractical for many departments, particularly smaller programs, due to budget constraints, competition with the technology sector, and administrative barriers in academic medical centers. This leaves a persistent gap: clinical physicists need modern, maintainable software tools but lack the time, training, or resources to develop them using conventional approaches.

Simultaneously, the landscape of software development has been undergoing a fundamental transformation driven by advances in generative artificial intelligence (AI). Large language models (LLMs) have demonstrated remarkable capabilities in code generation, enabling a paradigm that has been termed “vibe coding”, a phrase proposed to describe a workflow in which software is built primarily through natural language prompting rather than manual code authorship. In this paradigm, the developer describes desired functionality in plain language, and the AI generates the corresponding implementation, including the full technology stack, user interface components, and application logic. The developer's role shifts from writing code to specifying requirements, reviewing AI‐generated plans, testing output, and providing iterative feedback, i.e. a process closer to product management than traditional programming.

While LLMs have attracted significant attention in radiation oncology for clinical applications including auto‐segmentation, treatment plan optimization, and clinical decision support,^8^, ^9^, ^10^, ^11^, ^12^, ^13^ their potential to transform how medical physicists develop operational and clinical support software has received comparatively little attention.^14^ The present work explores this underexamined application of generative AI in medical physics. We describe the use of an AI‐powered vibe coding to replace an obsolete MATLAB‐based radioactive seed inventory management system with a modern application by a physicist with no prior web development experience. We detail a structured “Plan‐Review‐Execute‐Verify‐Feedback” development workflow, report quantitative development metrics, compare the resulting software against the legacy system, and discuss the broader implications of AI‐assisted development for the medical physics profession. To our knowledge, this represents the first published experience of vibe coding applied to the development of clinical medical physics software.

## 2 METHODS AND MATERIALS

### 2.1 Clinical context and requirements

#### 2.1.1 Seed inventory workflow

The brachytherapy program at our institution utilizes permanently implanted radioactive seeds across multiple clinical applications, including low‐dose‐rate (LDR) prostate implants using both iodine‐125 (I‐125) and palladium‐103 (Pd‐103) sources, I‐125 eye plaque treatments, GammaTile (GT Medical Technologies, Tempe, AZ) for post‐surgical brain tumor irradiation, and CivaSheet (CivaTech Oncology, Durham, NC) for intraoperative soft tissue applications. Managing the lifecycle of this diverse seed inventory is a critical operational and regulatory responsibility that requires meticulous record‐keeping. The seed inventory workflow encompasses several interdependent processes, each generating data that must be accurately captured and readily retrievable for clinical and regulatory purposes.

Each seed batch must be tracked through its full lifecycle. On receipt, the system records batch details and an in‐house well‐chamber calibration that verifies the vendor‐stated source strength, storing the chamber identity, calibration factor, and factor expiration date to maintain a complete chain of traceability. Batches are then held until implant, after which the system captures the final disposition of seeds, including those implanted, returned to inventory, or sent to radioactive waste, with waste deposits recorded by seed count, cumulative activity, and disposal date. Throughout, a current status is maintained for each batch (e.g., in stock, implanted, wasted), with summary views highlighting pending inventory and upcoming calibration‐factor expirations.

#### 2.1.2 Functional requirements

Prior to initiating development of the replacement system, the physics team conducted an internal needs assessment to define the functional requirements for the new seed inventory application. These requirements were informed by the limitations of the legacy system, feedback from clinical end‐users, and the regulatory and operational demands of the brachytherapy program. The key requirements are summarized in Table 1.

**TABLE 1:** Functional requirements for the replacement seed inventory system.

Requirement	Description	Availability in legacy software
Seed batch lifecycle management	Complete tracking of each seed batch from receipt through final disposition, including seed model, quantity, vendor activity, assay date, order number, patient assignment, implant date, and current status. Batch status transitions (e.g., “in stock” → “assigned” → “implanted” or “wasted”) enforced through a defined workflow to prevent illogical state changes.	Yes
Calibration record management	Storage of the full calibration record for each seed batch, including measured activity values, well chamber identity, chamber calibration factor, and calibration factor expiration date. Automated alerts when a chamber calibration factor is approaching or has exceeded its expiration date.	Partial
Comprehensive audit logging	Tamper‐resistant activity log recording every operation (data entry, record modification, status changes, deletions) with user identity, timestamp, and nature of the change. Log must be viewable by authorized users and not editable through the application interface.	No
Waste deposit tracking	Detailed record of all seeds transferred to radioactive waste, including number of seeds, activity at time of disposal, and disposal date, to support institutional radiation safety reporting and NRC compliance.	Using separate spreadsheet
Data migration from legacy system	Capability to import historical seed inventory records from the existing MATLAB‐based system to ensure continuity of the institutional record and avoid loss of historical data.	N/A
Record‐and‐verify system integration	Side‐by‐side view of open seed orders from Aria (Varian Medical Systems, Palo Alto, CA) within the inventory application, enabling verification of seed batch assignments against the treatment plan without launching Aria separately — reducing transcription errors and eliminating a time‐consuming manual cross‐referencing step.	No
Role‐based access control	Password‐protected access to system settings, calibration parameters, and administrative functions. General users may view records and perform routine data entry; configuration changes restricted to designated administrators.	No
Modern, intuitive user interface	Clean, well‐organized interface with logical navigation, clear visual hierarchy, and workflow guidance following contemporary UI/UX conventions to minimize the learning curve and improve adoption.	No

### 2.2 Development environment and AI tools

All software development was performed using Claude Code (Anthropic, San Francisco, CA), an agentic command‐line coding tool that operates within the developer's terminal and is powered by the Sonnet 4.6 large language model. Functioning as an autonomous agent, it reads the project codebase, plan multi‐step tasks, generates and edits files, executes terminal commands, and iterates on its own output in response to natural language feedback. The physicist performed no manual code authorship at any point, serving in a supervisory role of reviewing, testing, and directing, while the tool selected the technology stack, wrote the source code, and resolved errors. More details about technology stack, hardware and operating environment, version control, data privacy and intellectual property can be found in the Supplementary document.

### 2.3 Development methodology

The development process followed a structured, iterative workflow that we refer to as the “Plan‐Review‐Execute‐Verify‐Feedback” (PREVF) cycle. This cycle governed every feature addition and bug fix throughout the project, serving as the primary mechanism through which the physicist maintained control over the AI‐generated codebase. We emphasize that PREVF is not presented as a novel methodology. Rather, PREVF is offered as a practical, descriptive framework that adapts these established iterative principles to the specific context of AI‐assisted coding by a clinical physicist who is not a trained software engineer, and that formalizes the physicist's supervisory role of specifying, reviewing, testing, and steering the AI's output. Its contribution is therefore as an organizing description of a human‐in‐the‐loop workflow, not as a methodological innovation.

#### 2.3.1 The PREVF cycle

**FIGURE 1:**
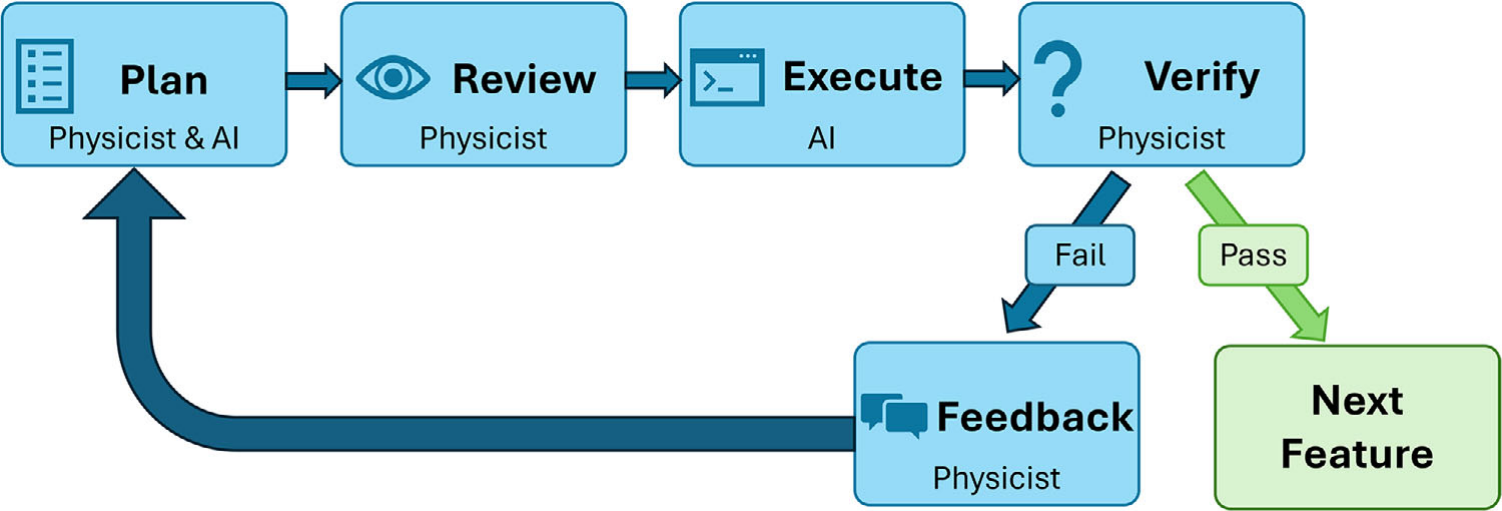
Schematic of the plan‐review‐execute‐verify‐feedback (PREVF) development cycle. Each box indicates the primary actor (Physicist, AI, or both).

Each development cycle consisted of five sequential phases as shown in Figure 1:

**Plan**. The physicist described the desired feature in natural language, then invoked Claude Code's built‐in plan command to generate a structured, step‐by‐step implementation plan without yet executing it.

**Review**. The physicist reviewed the plan for clinical correctness and alignment with the intended workflow, a step that did not require reading source code since the plan was expressed in plain language. Errors or missed requirements were corrected through natural‐language feedback, and the process advanced only after the physicist explicitly approved the plan.

**Execute**. Upon approval, AI autonomously implemented the plan, i.e. writing new code, modifying existing files, installing dependencies, and configuring the build system as needed. The physicist observed the execution but did not intervene in the coding process itself.

**Verify**. The physicist tested each new feature from the perspective of a clinical end‐user. Testing was manual and functionally oriented; no automated test suites were employed.

**Feedback**. If verification revealed bugs or deviations from the intended design, the physicist reported the issue back in plain language, or pasted application error messages directly into the terminal. Claude Code then diagnosed the issue and proposed a fix, beginning a new cycle that continued until the feature passed verification.

#### 2.3.2 Development progression

The overall development followed a two‐phase progression. In the initial phase, the physicist provided a comprehensive description of the application's core purpose and primary features in a single extended prompt. AI generated the foundational application structure in one execution cycle. This produced a working prototype with basic functionality within approximately 15 min.

Following this initial phase, development transitioned to an incremental, single‐feature approach. Each subsequent feature was implemented through its own dedicated PREVF cycle. The physicist tested each feature thoroughly before proceeding to the next, ensuring that new additions did not introduce regressions in previously completed functionality. This disciplined approach prevented the accumulation of compounding errors that could arise from implementing multiple untested features simultaneously.

When a new PREVF cycle introduced a bug in a previously working feature, the physicist reported the regression to Claude Code, which had access to the full project codebase and Git history. In most cases, the AI was able to identify and resolve the regression within a single feedback iteration. In rare instances where a fix proved elusive, the physicist used Git version control to roll back to the last known working state and re‐attempted the feature with a revised prompt that provided additional context or constraints.

### 2.4 Evaluation metrics

The performance of the AI‐driven development approach was evaluated across four dimensions: development efficiency, feature completeness, usability, and code maintainability.

#### 2.4.1 Development efficiency

Development effort was quantified using three metrics. First, total development time was recorded as both calendar days from project initiation to clinically deployable version and estimated active development hours (i.e., time spent actively prompting, reviewing, and testing, excluding idle time and clinical duties). Second, the total number of PREVF cycles (Section 2C) was counted across the entire project, providing a measure of iterative effort and a proxy for the complexity of the human‐AI interaction required. Third, the total lines of code in the final application were tallied to characterize the scale of the software produced.

#### 2.4.2 Feature completeness

Feature completeness was assessed by systematic comparison of the final application against the eight functional requirements defined prior to development (Table 1). Each requirement was classified as fully implemented, partially implemented, or not implemented based on functional testing of the deployed application.

#### 2.4.3 Usability

To evaluate the usability improvement relative to the legacy MATLAB system, a structured user survey was administered to physicists and staff in the brachytherapy group who had experience with both the old and new systems. The survey employed the System Usability Scale (SUS), a validated and widely used ten‐item questionnaire that yields a composite score from 0 to 100, with scores above 68 considered above average usability.^15^, ^16^ Each respondent completed the SUS independently for both the legacy system and the new application, which were concurrently available and operational at the time of the evaluation. In addition to the SUS, respondents were asked to rate the ease of performing five representative clinical tasks, i.e. registering a new seed batch, recording a calibration measurement, assigning seeds to a patient, generating a waste disposal record, and locating a historical batch record, on a five‐point Likert scale (1 = very difficult, 5 = very easy) for both systems. The task‐specific ratings complement the SUS by providing granular insight into which aspects of the workflow benefited most from the redesign.

The number of respondents (*N =* 5) was limited by the size of the brachytherapy physics group. Given this small sample, results are reported descriptively (median and range) rather than with inferential statistics.

#### 2.4.4 Code quality and maintainability

Code quality was characterized using a post‐hoc static analysis of the codebase, including codebase scale (lines of code and source file count), ESLint static analysis findings, and cyclomatic complexity; the detailed results are provided in the Supplementary Material (Table S1, Table S2 and Table S3). In addition, maintainability was evaluated qualitatively through a practical demonstration: team members who had not participated in the original development was asked to implement a minor feature modification using Claude Code, following the same PREVF workflow. The modifications are listed in the Supplementary Material. The ability of a non‐developer to successfully modify the application without assistance from the original author serves as a functional test of the claim that AI‐assisted development resolves the maintenance bottleneck characteristic of legacy in‐house tools. The nature of the requested modification, the time required, and the number of PREVF cycles needed are reported in the Results.

## 3 RESULTS

### 3.1 Development effort

All eight requirements defined in Table 1 were fully implemented in the final application, as confirmed by functional testing. The complete seed inventory application was developed over a calendar span of approximately nine active days. The total active development time defined as time spent prompting, reviewing plans, testing features, and providing feedback within Claude Code, was approximately 13 hours. This represents the cumulative time the physicist was actively engaged in the PREVF cycle.

We did not attempt a controlled comparison against traditional manual development, as developing equivalent functionality conventionally would require proficiency in web technologies (Electron, React, JavaScript) that the physicist did not possess, making a direct head‐to‐head comparison infeasible. Published descriptions of team‐based in‐house clinical software development in radiation oncology provide context for the substantial time and personnel typically involved in such efforts.^7^, ^17^

A total of approximately 115 natural language prompts were issued to Claude Code over the course of development. These prompts include feature requests, plan reviews, bug reports, UI refinement instructions. The 115 prompts corresponded to multiple PREVF cycles, as individual features frequently required several iterations of feedback and correction before passing verification.

The average prompting rate was approximately 8–9 prompts per active hour. This rate reflects the iterative, conversational nature of the vibe coding workflow: rather than composing lengthy, precisely specified requirements documents, the physicist issued short, focused prompts and relied on rapid feedback cycles to converge on the desired behavior.

### 3.2 Usability test

#### 3.2.1 Visual comparison

**FIGURE 2:**
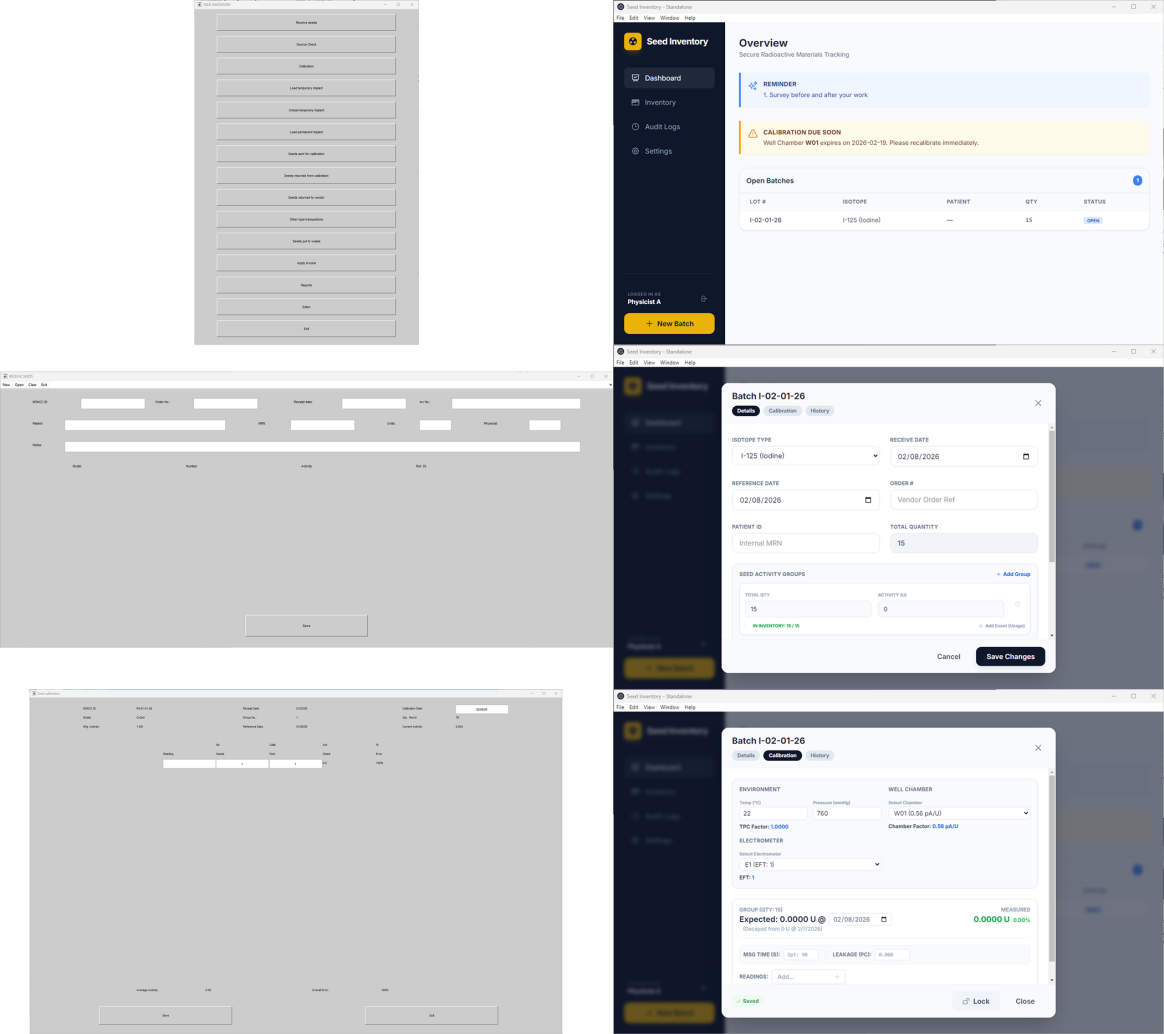
side‐by‐side comparison of legacy MATLAB interface (left) and new AI‐developed application (right) including representative screens for: main dashboard / home view, seed batch list and detail view, and calibration data entry (from top to bottom).

A side‐by‐side comparison of the legacy MATLAB interface and the new AI‐developed application is presented in Figure 2. The left column displays representative screens from the legacy system, characterized by small, non‐optimally sized fonts, densely packed text‐based button layouts, and cluttered data entry forms with minimal visual hierarchy. The right column displays the corresponding screens from the new application, featuring a clean dashboard with color‐coded status indicators and actionable reminders, well‐organized data tables with sortable columns and search functionality, and intuitive modal dialog windows for seed batch management and calibration data entry. The contrast illustrates the degree to which the AI‐generated interface adheres to contemporary UI/UX conventions such as consistent spacing, clear typography, logical grouping of related functions, and a navigation structure that guides users through the clinical workflow, despite the physicist having provided no explicit design specifications beyond the requirement for a “modern, intuitive” interface (Table 1).

#### 3.2.2 Usability survey

The legacy MATLAB system received a median SUS score of 32.5 (range: 5–55), while the new AI‐developed application received a median SUS score of 95 (range: 77.5–95). The median improvement of 62.5 points represents a shift from “Poor” to “Excellent” of rating by SUS norms, indicating a substantial improvement in perceived usability. Given the small sample (*N =* 5), these results are reported descriptively and should be interpreted as indicative rather than statistically definitive.

In addition to the SUS, respondents rated the ease of performing five representative clinical tasks on a five‐point Likert scale (1 = very difficult, 5 = very easy) for both systems. The results are summarized in Table 2.

**TABLE 2:** Task‐specific usability ratings (median, range) for the legacy and new systems.

Clinical Task	Legacy System	New Application
Register a new seed batch	1.5 (1–4)	5 (5–5)
Record a calibration measurement	1.5 (1–4)	5 (4–5)
Assign seeds to a patient	2.5 (1–4)	5 (5–5)
Generate a waste disposal record	2 (1–4)	5 (4–5)
Locate a historical batch record	1 (1–3)	5 (4–5)

Across all five tasks, the new application received higher median ratings than the legacy system. The largest improvements were observed for locating a historical batch record, which respondents noted had required the most navigation steps and trial‐and‐error in the legacy interface. Several respondents commented qualitatively that the new application felt comparable in usability to consumer‐grade software they use daily, whereas the legacy system typically required multiple training sessions before users felt comfortable, and even experienced users frequently needed to ask colleagues for guidance when performing less‐routine tasks.

### 3.3 Code maintainability

Team members who had not participated in the original development were asked to independently modify the application using Claude Code, without any assistance from the original developer. The results of the maintainability evaluation are summarized in Table 3.

**TABLE 3:** Maintainability evaluation: Independent modifications by non‐developer team members.

Task	Description	Type	Prompts	Time
1	Redesign calibration reading entry method	UI workflow change	2	< 10 min
2	Fix premature window closure on reading deletion	Bug fix	1	< 10 min
3	Add error notification for inaccessible data file	Error handling	1	< 10 min
4	Change activity decay equation	Logic modification	1	<10 min

All testers had minimal coding experience and no prior involvement in the development of the application. All tasks were completed successfully in under 10 min each, without any intervention or guidance from the original developer, and without the testers needing to read or understand the underlying source code. These results stand in direct contrast to the experience with the legacy MATLAB system, in which even minor modifications were infeasible without the original developer's involvement.

## 4 DISCUSSION

### 4.1 Comparison with traditional development approaches

The development timeline observed in this study stands in contrast to the conventional experience of building in‐house clinical physics software. Traditionally, a physicist who identifies an unmet software need must either learn the necessary programming skills, recruit a collaborator with software engineering expertise, or submit a request to an institutional IT department. A recent study advocating for dedicated software engineering support within medical physics teams acknowledged that even with a hired engineer, recruiting qualified candidates and navigating administrative barriers posed significant challenges.^7^ The vibe coding approach effectively bypasses these barriers by enabling the domain expert, i.e. the physicist who best understands the clinical requirement, to serve simultaneously as the product designer and development lead, with the AI functioning as the engineering team.

This is not to suggest that vibe coding eliminates the need for software engineering expertise entirely. Rather, it shifts the physicist's role from writing code to specifying requirements, reviewing AI‐generated plans, and validating output, which are skills that align more naturally with the analytical and quality assurance mindset that medical physicists already apply in clinical practice. The PREVF workflow described in this work formalizes this supervisory role and may serve as a template for other physics groups considering AI‐assisted development.

### 4.2 Implications for medical physics

Although this study was motivated by a brachytherapy‐specific problem, the same category of problem is encountered elsewhere in clinical medical physics. Medical physics departments routinely develop in‐house tools addressing clinical needs. Many of these tools share the same lifecycle vulnerabilities observed with the legacy system in this study: they are built by a single individual, poorly documented, difficult to maintain after that individual departs, and susceptible to obsolescence when operating system or platform changes break compatibility. The vibe coding approach demonstrated here may offer a practical option for modernizing these tools. We emphasize that this observation is limited to such operational tools and does not extend to applications involving dose calculation, plan optimization, or other physics‐critical computation, which were not evaluated in this study.

The direct cost of this approach was also modest. The entire development was completed using a consumer Claude Pro subscription (US $20 per month), with no additional usage‐based fees incurred. This is a small fraction of the cost of a commercial MATLAB license, and of engaging dedicated software engineering support. While a comprehensive cost‐benefit analysis comparing AI‐assisted development against alternatives such as hiring a part‐time developer or adopting commercial no‐code platforms is beyond the scope of this work, the low and transparent subscription cost supports the accessibility of this approach for resource‐constrained departments. Also note that these numbers reflect pricing at the time of development. The subscription tiers, usage‐based pricing, rate limits, and institutional or enterprise licensing terms may change substantially over the lifetime of a clinical application. Ongoing maintenance under this model depends on continued affordable access to a capable coding assistant, and departments should therefore treat current pricing as a planning estimate rather than a fixed long‐term cost.

### 4.3 Limitations

This study has several limitations that should be considered when interpreting the results.

First, the usability evaluation was conducted with a small sample (*N =* 5) drawn from a single department. Consequently, the SUS results are reported descriptively (median and range) rather than with inferential statistics, and the small sample size remains a limitation on the strength of the conclusions that can be drawn. While the SUS results are consistent with the qualitative improvements visible in the interface comparison, larger and more diverse user studies would be needed to draw definitive conclusions about usability gains.

ESLint findings and cyclomatic complexity measurements describe structural properties of the source code and do not establish functional correctness. They are not replacing independent expert code review, comprehensive verification and validation, or long‐term operational testing in the clinical environment. No third‐party software engineer reviewed the codebase, no automated test suite was implemented, and functional testing was performed manually by the developing physicist and by end users during clinical use. Sustained correctness, security, and dependency maintenance across a full software life cycle therefore remain unestablished, and departments adopting a similar approach should not treat static analysis metrics as evidence of clinical validation.

The results of this study are dependent on the capabilities of the specific LLM used (Claude Sonnet 4.6) at the time of development. LLM capabilities evolve rapidly, and the same prompts issued to a different model version or a competing product might yield different results. This introduces an element of non‐reproducibility that is atypical for conventional software development and should be acknowledged when reporting AI‐assisted development outcomes. We did not perform a controlled comparison across AI coding assistants. Such a comparison would require independently re‐developing an equivalent application with each model under matched prompting conditions and blinded evaluation of the resulting code, which constitutes a separate study beyond the scope of this single‐institution report. A systematic multi‐model benchmark is an important direction for future work.

This study focused on a relatively low‐risk and operational application within medical physics, while clinically important, does not involve physics‐intensive computation such as dose calculation algorithms, treatment plan optimization, or other tasks that require deep mathematical and physical reasoning in the source code. Whether LLM‐based code generation can reliably produce correct and efficient implementations for such computationally demanding and physics‐critical applications remains an open and important question. The successful development of an inventory management UI should not be extrapolated to assume equivalent feasibility for physics‐intensive software without dedicated investigation. Moreover, since developer may not fully understand at the source code level, it raises concerns for applications where software errors could have direct patient safety implications. Departments adopting this approach should carefully assess whether a given tool falls within an acceptable risk category for AI‐generated code, and should apply more rigorous validation and verification processes for higher‐risk applications.

Our evaluation addresses short‐term functional maintainability, namely the ability of a non‐developer to make targeted modifications through AI‐assisted prompting, but does not address long‐term software maintenance. Sustained maintainability over a software lifetime involves architectural understanding, regression control, dependency management, security updates, and ongoing support, none of which were evaluated here; the long‐term maintenance outcomes of AI‐generated clinical software therefore remain unknown. In addition, the new application was not benchmarked against any commercial or alternative seed inventory system, as the decision to build in‐house reflected the absence of a commercial product meeting our specific workflow requirements.

While the functional testing performed by the physicist and the independent maintainability evaluation provide confidence in the application's correctness for its intended use, these do not constitute the level of verification expected for software classified as a medical device or for tools that interface directly with treatment delivery systems. Departments considering broader adoption of vibe coding should develop internal guidelines for when formal QA processes are warranted.^17^

The present work is best understood as a single‐institution experience report rather than a generalizable evaluation. We do not claim broad generalizability; instead, we suggest that our experience may be informative for other departments facing similar challenges with developing in‐house software, and that the practical metrics and workflow reported here may help such groups assess whether a comparable approach is worth pursuing in their own setting. Establishing generalizability will require multi‐institution replication, in which physicists at different sites, with differing workflows, computing environments, and levels of prompting experience, independently attempt comparable development projects and report their outcomes. Such a study is an important direction for future work, alongside investigation of whether the approach extends to more complex, physics‐intensive software and the development of community best‐practice guidelines for AI‐assisted clinical software development, including appropriate levels of validation, code review, and disclosure.

## 5 CONCLUSION

Within the scope of this single‐institution experience, this study demonstrates that AI‐driven vibe coding enables the rapid development of a modern radioactive seed inventory application. The structured PREVF workflow maintained physicist oversight throughout the process, and the resulting software was independently modifiable by team members with minimal coding experience, helping to address the development and maintenance bottleneck that is pervasive among in‐house clinical physics tools. As LLM capabilities continue to advance, vibe coding has the potential to become a standard approach for medical physics departments seeking to modernize operational software without requiring dedicated software engineering resources, which is worth future investigation.

## AUTHOR CONTRIBUTIONS

Tonghe Wang and David Aramburu Núñez conceptualized the study. Tonghe Wang collected and analyzed data. Tonghe Wang prepared the initial manuscript. All authors participated in the discussion of results and revision of the manuscript.

## CONFLICT OF INTEREST STATEMENT

The authors declare no conflicts of interest.

## ETHICS STATEMENT

This research did not involve human or animal subjects and is exempt from ethics committee approval.

## Supporting information




**Supporting Information**: acm270795‐sup‐0001‐SuppMat.docx

## Data Availability

The data generated during and/or analyzed during the current study are available from the corresponding author upon reasonable request.

## References

[1] Wang X , Wang P , Li C , et al. An automated dose verification software for brachytherapy. J Contemp Brachytherapy. 2018;10(5):478‐482. doi:10.5114/jcb.2018.79396 30479626 PMC6251445

[2] Damato AL , Devlin PM , Bhagwat MS , et al. Independent brachytherapy plan verification software: improving efficacy and efficiency. Radiother Oncol. 2014;113(3):420‐424. doi:10.1016/j.radonc.2014.09.015 25458129

[3] Shirazi MAM , Faghihi R , Siavashpour Z , Nedaie HA , Mehdizadeh S , Sina S . Independent evaluation of an in‐house brachytherapy treatment planning system using simulation, measurement and calculation methods. J Appl Clin Med Phys. 2012;13(2):3687. doi:10.1120/jacmp.v13i2.3687 22402384 PMC5716415

[4] Kim Y , Sheng Y , Wang C , Lu K . Development of GUI‐based simple independent dose calculation software without DICOM data transfer for high‐dose‐rate brachytherapy. J Contemp Brachytherapy. 2024;16(6):457‐466. doi:10.5114/jcb.2024.146835 39943978 PMC11812136

[5] Simiele SJ , Chen X , Lee C , et al. Development and comprehensive commissioning of an automated brachytherapy plan checker. Brachytherapy. 2020;19(3):355‐361. doi:10.1016/j.brachy.2020.02.003 32249182

[6] Xu J , Zhang B , Guerrero M , Kalavagunta C , Chen S , Xu H . Technical note: an automated document verification tool in radiation oncology EMR: application for LDR prostate brachytherapy. J Appl Clin Med Phys. 2024;25(9):e14466. doi:10.1002/acm2.14466 39079544 PMC11492343

[7] Badawy MK , Carrion D . Strengthening medical physics through dedicated software engineering support. Physica Med. 2025;129:104874. doi:10.1016/j.ejmp.2024.104874 39642575

[8] Rajendran P , Yang Y , Niedermayr TR , et al. Large language model‐augmented learning for auto‐delineation of treatment targets in head‐and‐neck cancer radiotherapy. Radiother Oncol. 2025;205:110740. doi:10.1016/j.radonc.2025.110740 39855601 PMC11956750

[9] Rajendran P , Chen Y , Qiu L , et al. Auto‐delineation of treatment target volume for Radiation Therapy using Large Language Model‐Aided Multimodal Learning. Int J Radiat Oncol Biol Phys. 2025;121(1):230‐240. doi:10.1016/j.ijrobp.2024.07.2149 39117164 PMC13041240

[10] Wei S , Hu A , Liang Y , et al. Feasibility study of automatic radiotherapy treatment planning for cervical cancer using a large language model. Radiat Oncol. 2025;20(1):77. doi:10.1186/s13014-025-02660-5 40375332 PMC12083153

[11] Wang P , Liu Z , Li Y , et al. Fine‐tuning open‐source large language models to improve their performance on radiation oncology tasks: a feasibility study to investigate their potential clinical applications in radiation oncology. Med Phys. 2025;52(7):e17985. doi:10.1002/mp.17985 40665561 PMC12694766

[12] Hager P , Jungmann F , Holland R , et al. Evaluation and mitigation of the limitations of large language models in clinical decision‐making. Nat Med. 2024;30(9):2613‐2622. doi:10.1038/s41591-024-03097-1 38965432 PMC11405275

[13] Yalamanchili A , Sengupta B , Song J , et al. Quality of Large Language Model responses to radiation oncology patient care questions. JAMA Netw Open. 2024;7(4):e244630‐e244630. doi:10.1001/jamanetworkopen.2024.4630 38564215 PMC10988356

[14] Court LE , Smit J , Strauss L , et al. Leveraging large language models for heuristic usability assessment of medical software: insights with the Radiation Planning Assistant. J Appl Clin Med Phys. 2026;27(2):e70495. doi:10.1002/acm2.70495 41708070 PMC12916175

[15] Hyzy M , Bond R , Mulvenna M , et al. System Usability Scale benchmarking for Digital Health Apps: meta‐analysis. JMIR Mhealth Uhealth. 2022;10(8):e37290. doi:10.2196/37290 35980732 PMC9437782

[16] Brooke J . SUS: a retrospective. J Usability Studies. 2013;8(2):29‐40. numpages = 12.

[17] Moran JM , Paradis KC , Hadley SW , et al. A safe and practical cycle for team‐based development and implementation of in‐house clinical software. Adv Radiat Oncol. 2021;7(1):100768. doi:10.1016/j.adro.2021.100768 35071827 PMC8767245

