# Supplementary material for: AI‐assisted software development for brachytherapy seed inventory management
Source: J Appl Clin Med Phys. 2026 Sep 9;27(9):e70795. doi: 10.1002/acm2.70795 (PMC13556308; doi:10.1002/acm2.70795)
Supplement: Supplementary file 1 [file ACM2-27-e70795-s001.docx]

**Supplementary Material**

*A Single-Institution Experience Using AI-Assisted Software Development for Brachytherapy Seed Inventory Management*

**S1. Technology Stack**

The technology stack was selected by the AI tool based on the functional requirements communicated through natural language prompts; the physicist did not prescribe specific frameworks or libraries. The resulting architecture used Electron (OpenJS Foundation) as a cross-platform desktop application wrapper, React (Meta Platforms, Menlo Park, CA) for the front-end user interface, and a local JSON-based file storage system for data persistence. This combination enabled distribution as a single installable desktop package compatible with hospital workstations, provided a modern component-based UI, and ensured that all data remained on the local institutional network without requiring external servers, cloud connectivity, or database infrastructure.

**S2. Hardware and Operating Environment**

Development was performed on a standard hospital-issued workstation running Windows 11 (Microsoft, Redmond, WA). The same class of workstation was used for testing and deployment, ensuring compatibility with the target clinical computing environment. No specialized hardware, GPU resources, or cloud computing infrastructure was required for development — the AI processing occurred on Anthropic's servers via Claude Code's API connection, while all code compilation, testing, and execution were performed locally.

**S3. Version Control**

Source code was managed using Git (Software Freedom Conservancy), with a repository hosted on the institution's GitHub Enterprise instance. The repository was created under the department's organizational page, granting all group members read and contributor access. A detailed README file generated by AI was included in the repository to document the application's purpose, installation instructions, usage guidelines, and development workflow, ensuring that any group member could understand, deploy, and contribute to the project without reliance on the original developer, directly addressing the maintenance bottleneck that had plagued the legacy system. Version control provided a complete history of all code changes, enabling rollback to previous states if an AI-generated modification introduced regressions, and facilitating future maintenance by preserving the full evolution of the codebase.

**S4. Data Privacy and Intellectual Property**

Strict data privacy protocols were maintained throughout the development process. Because Claude Code operates by sending code context and natural language prompts to Anthropic's servers for LLM processing, careful attention was paid to ensuring that no Protected Health Information (PHI), patient identifiers, or clinical data were included in any prompt or code file processed by the AI. Only software logic, user interface code, and generic data structures (e.g., placeholder field names such as "patientID" and "seedModel") were transmitted; all real patient data was entered exclusively at runtime on the local workstation after the application was fully built and deployed.

The Anthropic Terms of Service in effect at the time of development were reviewed to confirm that the physicist and host institution retain full intellectual property (IP) ownership of all source code generated through Claude Code. This was an important institutional consideration, as the resulting software was intended for long-term clinical use and potential sharing with other brachytherapy programs.

# S5. modification tasks

Four modification tasks of varying complexity were assigned:

**Task 1 — UI workflow change.** A tester was asked to change the method of entering electrometer readings in the calibration module. This required modifying the data entry interface and adjusting how input values were captured and stored, a change that touched both front-end components and underlying data handling logic. The tester completed the modification successfully using 2 prompts.

**Task 2 — Bug fix.** A tester was asked to resolve a bug in which deleting a single calibration reading caused the entire calibration window to close unexpectedly, rather than simply removing the selected entry and keeping the window open. This required diagnosing an event handling error in the UI component. The tester identified and resolved the issue using 1 prompt.

**Task 3 — Error handling improvement.** A tester was asked to add a user notification when the application cannot access the locally stored seed inventory JSON file at startup. Previously, this failure condition caused the application to silently fail to launch, leaving the user with no indication of the problem. The tester implemented an informative error dialog that alerts the user and provides guidance on resolving the file access issue, using 1 prompt.

**Task 4 — Logic modification.** A tester was asked to modify the activity decay calculation used throughout the application, changing the implementation from $A = A₀\cdot exp(-0.693\cdot\frac{t}{T_{1/2}})$ to the form $A = A₀\cdot{0.5}^{\frac{t}{T_{1/2}}}$ to better match with the calculations from legacy system. This task required locating and updating the underlying calculation logic within the codebase and therefore involved physics-relevant computation rather than user interface changes or error handling, representative of the type of formula modification a physicist may need to make as clinical practices evolve. The modification was completed using 1 prompt.

# S6. Quantitative Code Quality Metrics

To characterize the quality of the AI-generated codebase, we performed a post-hoc analysis of the final application source code. The analysis covered three dimensions: codebase scale, static analysis findings, and cyclomatic complexity. All metrics were computed on the application source only, excluding third-party dependencies (node_modules), build artifacts (.vite and dist directories), configuration files, and test files unless otherwise noted. It should be noted that the project did not originally ship with a linting configuration; the static analysis reported below was conducted separately for the purpose of this evaluation and was not part of the original development workflow.

## S6.1. Codebase Scale

The final application comprised 7,698 lines of application code across 60 JavaScript and JSX source files. A breakdown by file category is provided in Table S1.

**Table S1.** Codebase scale by file category.

| **Category** | **Files** | **Lines** | **Included in metrics** |
| --- | --- | --- | --- |
| Application code (.js / .jsx) | 60 | 7,698 | Yes |
| Test code (*.test.js) | 24 | 2,377 | No |
| Configuration (*.config.js, postcss, tailwind) | 8 | 203 | No |
| CSS / HTML assets | 3 | 156 | No |

Notably, the AI tool generated a substantial test suite (24 test files comprising 2,377 lines of test code) alongside the application code without an explicit request from the physicist to implement automated testing, indicating that testing infrastructure was produced as part of the default development workflow.

## S6.2. Static Analysis

Static analysis was performed using ESLint version 9 with the recommended rule set augmented by complexity and unused-variable rules, with Node.js and Vite global variables declared to avoid spurious findings. The analysis was run against the non-test application source. A total of 123 findings were reported, comprising 8 errors and 115 warnings; no critical or correctness defects were identified. A breakdown by rule is provided in Table S2.

**Table S2.** ESLint findings by rule (non-test application source).

| **Rule** | **Count** | **Nature** |
| --- | --- | --- |
| no-unused-vars | 86 (warning) | Unused imports and destructuring throwaways |
| complexity (> 10) | 29 (warning) | Large coordinator functions (see Table S3) |
| no-empty | 7 (error) | Intentional silent-skip catch blocks for optional integrations |
| react-hooks/exhaustive-deps | 1 (error) | One missing hook dependency |
| *Total* | *123* | *8 errors, 115 warnings; no correctness defects* |

The 8 errors were all minor maintainability items: 7 empty catch blocks used intentionally to silently skip optional integrations, and 1 missing React hook dependency. None represented functional or correctness defects. The 115 warnings were dominated by unused variables arising from import and destructuring patterns, together with functions exceeding the default cyclomatic complexity threshold.

## S6.3. Cyclomatic Complexity

Cyclomatic complexity was assessed using the ESLint complexity rule with a threshold of 10. Twenty-nine functions exceeded this threshold. The highest-complexity functions are listed in Table S3. The complexity outliers were concentrated in a small number of large state-coordinating components (App.jsx, SeedModal/index.jsx, and SettingsView.jsx) and in the legacy data import parser (legacyImporter.js). The remaining functions above threshold fell within the moderate range of 11 to 18, indicating that elevated complexity was localized to a few inherently complex coordinator modules rather than spread broadly across the codebase.

**Table S3.** Functions with the highest cyclomatic complexity (CC).

| **CC** | **Location** |
| --- | --- |
| 44 | src/renderer/components/SettingsView.jsx |
| 40 | src/renderer/components/SeedModal/index.jsx |
| 36 | src/main/legacyImporter.js |
| 34 | src/renderer/App.jsx |
| 24 | src/renderer/components/SeedModal/tabs/CalibrationTab.jsx |
| 20 | src/main/storage/sanitize.js |
| 20 | src/renderer/components/ChatPanel.jsx |
| 19 | src/main/integrations/ollama.js |

## S6.4. Interpretation and Caveats

These metrics indicate that the AI-generated codebase was free of correctness-level static analysis defects and exhibited complexity that was concentrated in a small number of expected coordinator modules. The findings should be interpreted with appropriate caveats. Static analysis and cyclomatic complexity characterize code structure and maintainability indicators, but they do not constitute a comprehensive software quality assurance process; they do not assess functional correctness, security posture, or compliance with formal medical software development standards (e.g., IEC 62304). As discussed in the main text, more rigorous validation and verification would be advisable before broader distribution of the software, particularly for any application that could influence clinical decision-making or treatment delivery.
